# Supplementary material for: NMR-Based Metabolomic Approach Tracks Potential Serum Biomarkers of Disease Progression in Patients with Type 2 Diabetes Mellitus
Source: J Clin Med. 2019 May 21;8(5):720. doi: 10.3390/jcm8050720 (PMC6571571; doi:10.3390/jcm8050720)
Supplement: Supplementary file 1 [file jcm-08-00720-s001.pdf]

Figure S1. PLSDA score scatter plot between CG and T2DM-NC

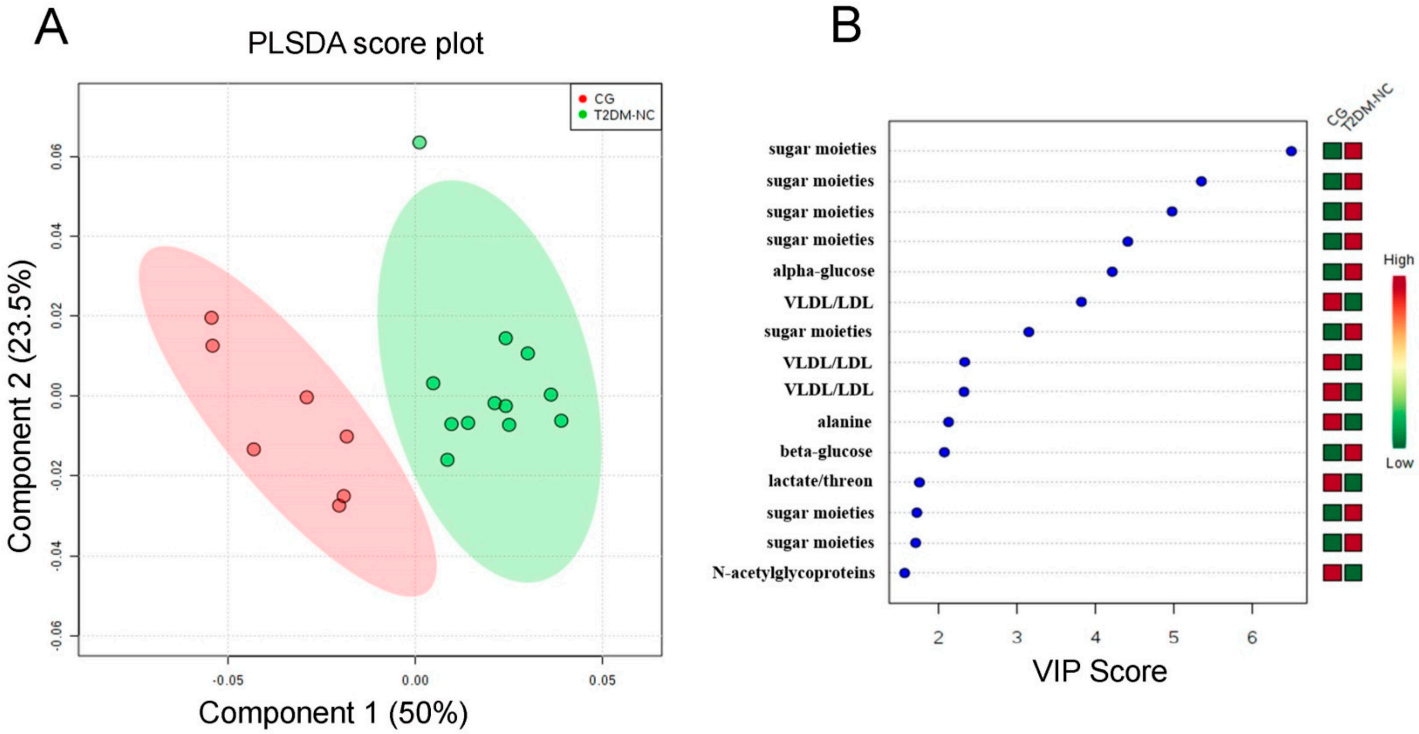

Figure S2. PLSDA score scatter plot between CG and T2DM-C

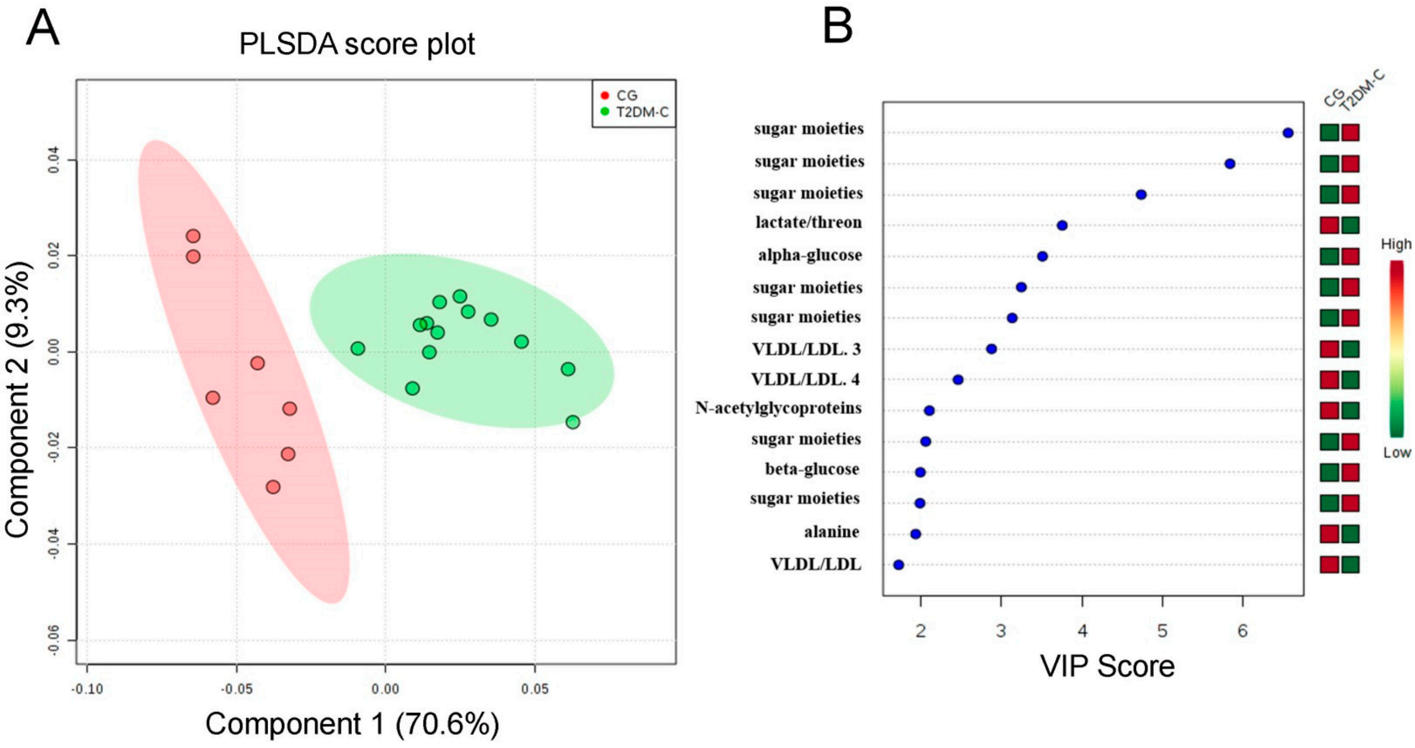

Figure S3. PLSDA score scatter plot between T2DM-C and T2DM-NC

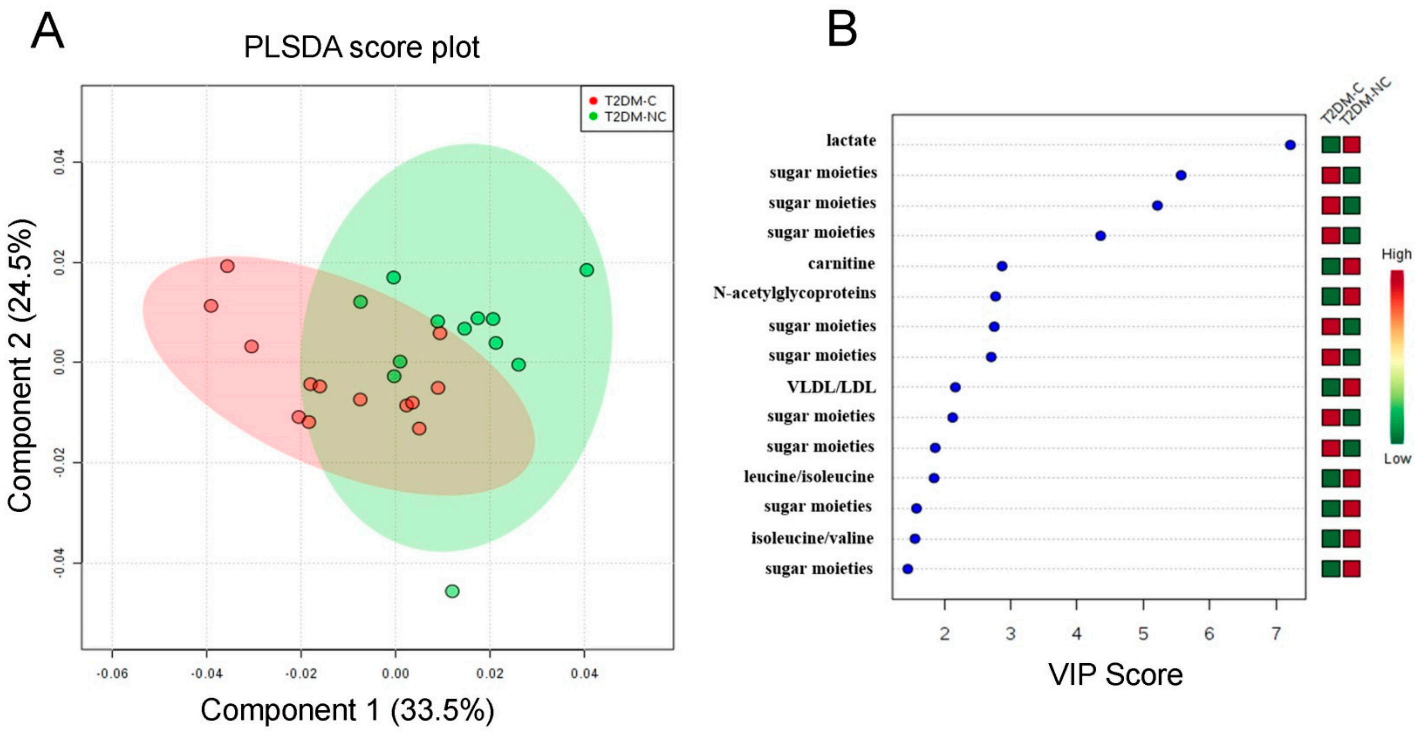

Table S1. Results from Pathway Analysis with MetPA for serum samples of CG *vs* T2DM-NC patients.

Pathway Name	Matched metabolites	p-value	impact
Alanine, aspartate and glutamate metabolism	Alanine, Glutamine, Glutamate (3/24)	$1.055*10^{-4}$	0.44065
Aminoacyl-tRNA biosynthesis	Phenylalanine; Glutamate; Glutamine; Methionine; Valine; Alanine; Lysine; Isoleucine; Leucine; Tyrosine (10/75)	$3.472*10^{-4}$	0.11268
Lysine degradation	Lysine (1/47)	$5.174*10^{-4}$	0.14675
D-Glutamine and D-glutamate metabolism	Glutamate; Glutamine (2/11)	$6.9113*10^{-4}$	0.13904
Starch and sucrose metabolism	α-, β-glucose (2/50)	$3.1103*10^{-8}$	0.03195
Glycolysis or gluconeogenesis	α-, β-glucose; lactate (3/31)	$3.0189*10^{-8}$	0.01094

Total number of compounds involved in each pathway and metabolites actually matched from the uploaded data; p is the original p-value calculated from the enrichment analysis; the impact is the pathway impact value calculated from pathway topology analysis.

Table S2. Results from Pathway Analysis with MetPA for serum samples of CG *vs* T2DM-C patients.

Pathway Name	Matched metabolites	p-value	impact
Alanine, aspartate and glutamate metabolism	Alanine, Glutamine, Glutamate (3/24)	$1.2835*10^{-7}$	0.44065
Aminoacyl-tRNA biosynthesis	Phenylalanine; Glutamate; Glutamine; Methionine; Valine; Alanine; Lysine; Isoleucine; Leucine; Tyrosine (10/75)	$3.9568*10^{-8}$	0.11268
Lysine degradation	Lysine (1/47)	$8.3129*10^{-7}$	0.14675
D-Glutamine and D-glutamate metabolism	Glutamate; Glutamine (2/11)	$4.6644*10^{-6}$	0.13904

Total number of compounds involved in each pathway and metabolites actually matched from the uploaded data; p is the original p-value calculated from the enrichment analysis; the impact is the pathway impact value calculated from pathway topology analysis.

Table S3. Results from Pathway Analysis with MetPA for serum samples of T2DM-C *vs* T2DM-NC patients.

Pathway Name	Matched metabolites	p-value	impact
Alanine, aspartate and glutamate metabolism	Alanine, Glutamine, Glutamate (3/24)	0.13152	0.44065
D-Glutamine and D-glutamate metabolism	Glutamate; Glutamine (2/11)	0.016616	0.13904
Lysine degradation	Lysine (1/47)	0.008533	0.14675
Phenylalanine metabolism	Phenylalanine; tyrosine (2/45)	0.006458	0.11906
Aminoacyl-tRNA biosynthesis	Phenylalanine; Glutamate; Glutamine; Methionine; Valine; Alanine; Lysine; Isoleucine; Leucine; Tyrosine (10/75)	0.00438	0.11268
Valine, leucine and isoleucine biosynthesis	Valine; Isoleucine; Leucine (3/27)	$5.1516*10^{-4}$	0.03975
Valine, leucine and isoleucine degradation	Valine; Isoleucine; Leucine (3/40)	$5.1516*10^{-4}$	0.02232

Total number of compounds involved in each pathway and metabolites actually matched from the uploaded data; p is the original p-value calculated from the enrichment analysis; the impact is the pathway impact value calculated from pathway topology analysis.
